# Patient-reported symptoms in the detection of head and neck cancer recurrence: a systematic review

**DOI:** 10.3389/fonc.2025.1632592

**Published:** 2025-06-18

**Authors:** Kate Hulse, Rhona Hurley, Anja Lowit, Roma Maguire, Claire Paterson, Catriona M. Douglas

**Affiliations:** ^1^ Department of Psychological Sciences and Health, University of Strathclyde, Glasgow, United Kingdom; ^2^ Glasgow Head and Neck Cancer (GLAHNC) Research Group, Glasgow, United Kingdom; ^3^ School of Medicine, Dentistry and Nursing, University of Glasgow, Glasgow, United Kingdom; ^4^ Strathclyde Institute of Pharmacy and Biomedical Sciences, University of Strathclyde, Glasgow, United Kingdom; ^5^ Department of Computer and Information Sciences, University of Strathclyde, Glasgow, United Kingdom; ^6^ Beatson West of Scotland Cancer Centre, Glasgow, United Kingdom; ^7^ Department of Otolaryngology – Head and Neck Surgery, Glasgow Royal Infirmary/Queen Elizabeth University Hospital, Glasgow, United Kingdom

**Keywords:** head and neck cancer, cancer morbidity, cancer recurrence, patient reported outcome (PRO), symptomatic recurrence

## Abstract

**Introduction:**

Patient-initiated follow-up (PIFU) after treatment for head and neck cancer (HNC) relies on the signs and symptoms of recurrence being detectable by patients. We examine the evidence for patient-reported symptoms as an indicator of recurrence.

**Methods:**

A search was conducted via OvidMEDLINE and Embase (2010 to January 2024) plus sources of grey literature for studies which describe patient-reported symptoms and recurrent disease. Findings are reported as per PRISMA guidelines.

**Results:**

Twenty studies were included which were highly heterogenous. The median sensitivity of patient-reported symptoms to detect recurrence is 47.3%. Median specificity, positive-predictive value (PPV) and negative-predictive value (NPV) were 79.3%, 9.3% and 98.0% respectively. New symptoms were generally reported at routine follow-up rather than expedited appointments.

**Conclusion:**

The high specificity and NPV of patient-reported symptoms means recurrence is unlikely in the absence of symptoms. Patient education and collection of prospective data through digital health technologies may increase the effectiveness of PIFU.

## 1 Introduction

Head and neck cancer (HNC) is the 8^th^ most common cancer in the UK (1). Following treatment, over a third of patients experience recurrence depending on the tumour stage and primary site. Treatment options for recurrent disease include salvage surgery, re-irradiation, palliative chemotherapy and/or immunotherapy. A recent meta-analysis reported 5-year overall survival following salvage surgery between 26-67% (2). Salvage treatments generally carry significant morbidity but are more likely to be successful if recurrence is detected at an earlier stage (3) and therefore the emphasis remains on early identification.

The rationale for follow-up after HNC treatment is the detection of recurrent disease and the management of post-treatment toxicity. Current UK recommendations are for patients to be seen at least every 2 months for the first 2 years, followed by every 3–6 months for a minimum of 5 years in total. It is recommended that patients should have clinical examination at every follow-up including, when appropriate, nasopharyngolaryngoscopy (4). Patient-initiated follow-up (PIFU) is not currently routine practice, and many patients prefer a scheduled follow-up approach for reassurance and reliable access to information (5). PIFU has been mooted in HNC since there is limited evidence that regular follow-up impacts survival outcomes and outpatient capacity can struggle to meet the demand of recommended appointment frequency. A recent systematic review of PIFU following treatment for other cancer types found similar rates of recurrence, survival, quality of life, fear of recurrence and patient satisfaction in breast and colorectal cancer compared to conventional follow-up. However, it is noted that all breast cancer PIFU programmes included regular mammograms and colorectal PIFU programmes included either regular testing for faecal occult blood or CT scans (6).

For PIFU to replace the function of routine surveillance in HNC, it should be a reliable tool to identify recurrence; this has not yet been demonstrated but is the subject of on-going research (7). In this systematic review, we aim to examine the value of patient-reported symptoms in the detection of recurrence and second primary (SP) in HNC and therefore the potential role of symptoms in PIFU.

## 2 Methods

### 2.1 Eligibility criteria

#### 2.1.1 Inclusion

- Studies describing patient-reported signs or symptoms after curative treatment for Head and Neck squamous-cell carcinoma (HNSCC),
- Some or all the patients in the cohort received primary cancer treatment after January 2010,
- Studies report rates of recurrence and/or SP detection in relation to patient symptoms,
- Full text available in English.

#### 2.1.2 Exclusion

- Study subjects have known recurrent or metastatic HNSCC, non-SCC histology, cutaneous, upper oesophageal cancer or thyroid cancer,
- Patients receiving palliative or non-curative treatment,
- Case reports,
- No original data presented e.g. review articles.

### 2.2 Information sources

The search was conducted on OvidMEDLINE (1974 to January 26 2024) and Embase (1946 to January 26 2024). Sources of grey literature were searched via four channels: the online repository Open Access Thesis and Dissertations (oatd.org), ClinicalTrials.gov, MedRxiv, and a Google search where the first 100 hits were screened for relevance. The references and citations of included studies were also subject to screening followed by full-text review if deemed relevant.

### 2.3 Search strategy

This review was registered on PROSPERO (CRD42024510566) and reported according to PRISMA guidelines (8). OvidMEDLINE and Embase were searched separately using the following terms: (head and neck cancer) AND (patient reported or patient-reported or symptom*) AND (recurrent or recurrence or second primary). “Head and neck cancer” as subject/keyword and “recurrence” were used to search the oatd.org database with a filter for English-language. The search terms are described in detail and for other sources in **Appendix 1**.

### 2.4 Selection process

Duplicates were manually removed by screening of the title, first author name and year of publication. Abstracts were screened for inclusion or exclusion by two authors (KH and RH) according to the criteria above and full-text review was performed with over-sight by CD who made a final decision on inclusion in cases of disagreement.

A cut-off of treatment prior to 2010 was applied since the wide-spread adoption of intensity-modulated radiotherapy (IMRT) around this time reduced treatment-related long-term sequelae (9). Similarly, the role of human-papilloma virus (HPV) in oropharyngeal cancer was recognised and changed the understanding of risk and recurrence related to these cancers (10).

### 2.5 Data collection

Data was extracted by KH. Data items retrieved were the first author, country of study, year of publication, number of patients included in study, period of patient treatment, demographic and clinical characteristics of patients, the patient-reported outcome measure used, and key findings related to recurrence of disease or SP.

### 2.6 Effect measures

Data was collected on the number of true positives (patients with reported symptoms and confirmed recurrence), true negatives (asymptomatic patients without recurrence), false positives (symptomatic without recurrence) and false negatives (asymptomatic with recurrence). Where sufficient data was reported a calculation of sensitivity, specificity, positive-predictive value (PPV) and negative predictive value (NPV) was performed. Confidence intervals of 95% were calculated using RStudio 2024.12.0.

### 2.7 Synthesis methods

Variables as described above were tabulated. A narrative synthesis of results was performed. Studies were almost exclusively observational in nature with heterogenous study populations and study design therefore meta-analysis could not be meaningfully performed.

### 2.8 Quality assessment and risk of bias

Most of the studies were a cohort study involving longitudinal observation of a group of patients following treatment and assessment of their outcomes. There are no recommended risk-of-bias tools specific to this study design. The following sources of potential bias were assessed, derived from the Newcastle-Ottawa Scale (NOS) for assessing the quality of nonrandomised studies (11): 1) representativeness of the population, 2) method of assessing symptoms, 3) adequacy of follow-up and 4) identification of and control for potential confounding factors.

## 3 Results

### 3.1 Study selection

**Figure 1:**
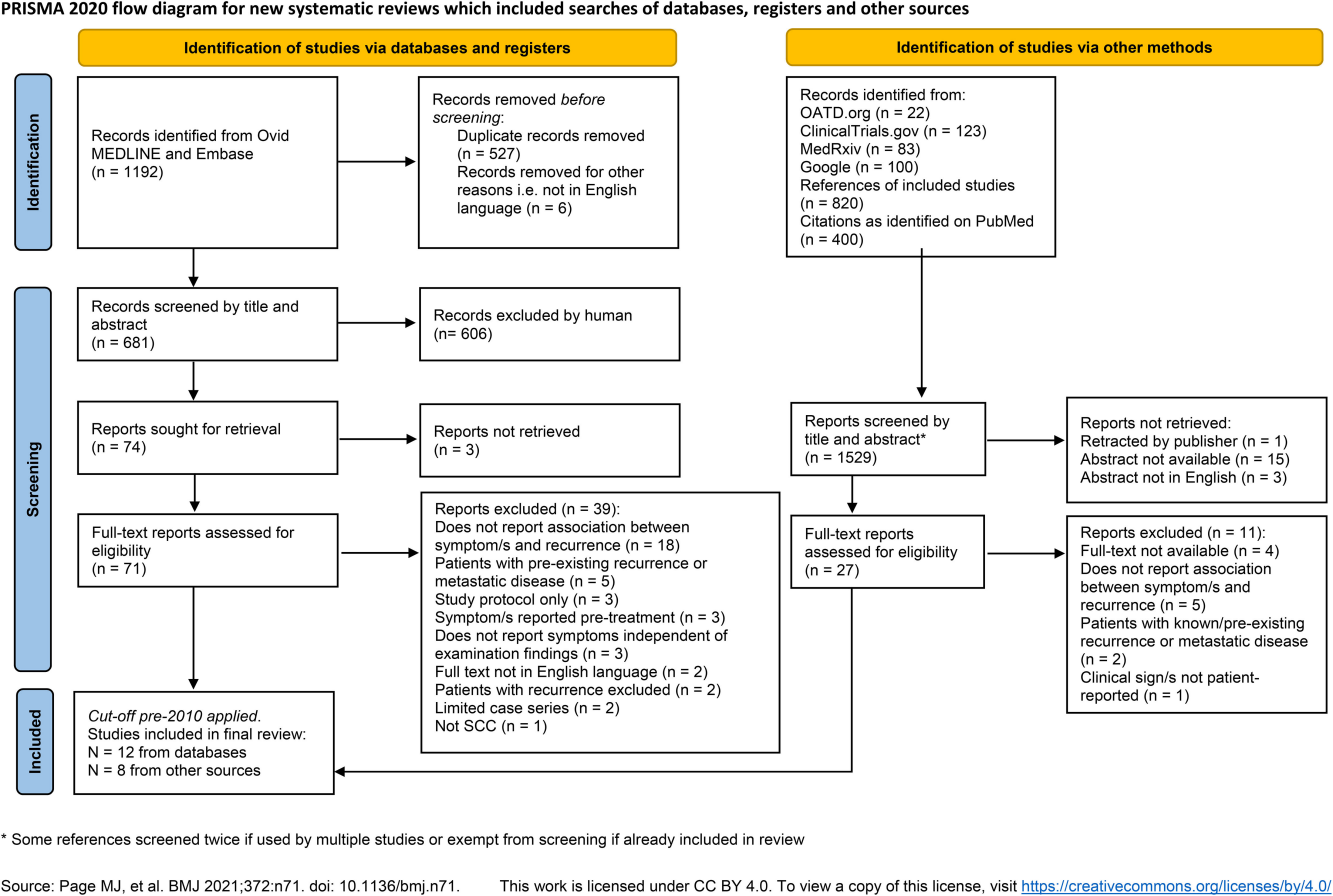
PRISMA flow diagram shows the data sources, excluded records and reasons for exclusion. *Some references screened twice if used by multiple studies or exempt from screening if already included in review. Source: Page MJ, et al. BMJ 2021;372:n71. doi: 10.1136/bmj.n71. This work is licensed under CC BY 4.0. To view a copy of this license, visit https://creativecommons.org/licenses/by/4.0/.

The search of OvidMEDLINE and Embase yielded a total of 1192 results. After de-duplication there were 887 records remaining. Six articles were removed as the publication was not available in English language and a further 213 were removed as the full text was not available e.g. conference abstract only. The remaining 668 records were reviewed by KH and RH for eligibility. Twenty-two theses were also screened for inclusion and one dissertation was sought for full-text review. ClinicalTrials.gov search yielded 123 records which were screened for relevance, but none sought for full-text review. MedRxiv found 83 records, 1 sought for full-text review. A Google search did not yield any previously unfound records. The cut-off for treatment time prior to 2010 was applied to the final set of records since it is not possible to search or filter for this in the conventional way. The process of study selection is shown in the PRISMA flow-diagram (**Figure 1**).

### 3.2 Study characteristics

A total of 20 studies are included in this review, published between 2013-2024. Key features and results of individual studies can be found in **Table 1** (12–31). The study populations represent a heterogeneous group of patients with different primary disease site and stages and varying treatment modalities. There are 17 cohort studies, of which 4 are retrospective. In these studies, the rate of symptomatic and asymptomatic recurrences or SP are generally reported however few state the rate of patient-reported symptoms in the whole cohort. There were 3 case series, of which 2 assessed a patient group which were all symptomatic.

**Table 1:** Summary of included studies.

Author	Country	Year of publication	N	Period of treatment	Tumour primary site and stage	Primary treatment	Nature of patient-reported symptom/s	Key finding/s related to symptomatic recurrence or second primary
Belcastro	USA	2021	89	2010-2019	Oropharyngeal SCC (50.6% HPV-positive). 16.9% stage III/IV.	Surgical treatment +/- adjuvant CRT (rates NS)	New throat pain or otalgia	32 (36%) patients had a recurrence/SP. Significantly more patients had throat pain and/or otalgia when presenting with a second or recurrent oropharyngeal cancer compared to primary cancer - 23/32 (71.9%) and 15/57 (26.3%) respectively. On multivariate logistic regression, simultaneous throat pain and otalgia was a significant predictor of recurrence.
Blatt	Germany	2022	760	2000-2015	All oral SCC. Stage NS, 198 (29%) T3/4.	Primary surgery	Self-reported symptoms, not pre-defined	216 (28.4%) patients had a recurrence, of which 18 (8%) were detected via self-reported symptoms. Symptoms included pain, burning sensation, difficulty chewing and swallowing and globus sensation; frequency of symptoms not stated.
Brands	Netherlands	2022	307	2006-2012	All oropharynx, 155 (50.5%) HPV PCR or P16-positive. 250 (81%) stage III/IV.	RT alone 187 (61%), CRT 81 (26%), surgery + RT/CRT 30 (9.8%)	Routine or interval visit and whether patient-initiated plus “potential signs and symptoms of new disease”	81 (26.4%) locoregional recurrence or SP of which 69 (85.2%) were symptomatic. 100% of SP tumours were symptomatic. OS not significantly different by means of detection.
Daga	India	2021	700, of which 189 FU	FU 2020	Whole cohort (including new cancer cases) 554 (80%) oral cavity. 623 (89.9%) stage III/IV.	NS	Symptoms inc. pain, ulceration, swelling and weight loss	81 (43%) of FU patients were symptomatic of which 12 (14.8%) had recurrence. Rate of recurrence in asymptomatic patients not given.
Ellis	UK	2021	5123	FU 2017-2018	1845 (36%) oropharynx, 1151 (22.5%) glottic and 527 (10.3%) oral cavity most common subsites. Stage NS, 1781 (34.8%) T3/4.	2123 (41.4%) surgery to primary site, 2756 (53. 8%) RT +/- CT. 1661 (32.4%) neck dissection and/or 3132 (61.1%) RT to at least one neck.	New symptoms since previous consultation, not pre-defined	122 patients had recurrence or SP, and an additional 50 patients had residual disease (within 6 months). Of all confirmed malignancy, 86 (50%) were asymptomatic. 1213 (23.6%) patients developed new symptoms. Difficulty breathing had the highest PPV for recurrence (16.2%), followed by pain in the throat/mouth (10.4%), pain in the neck/shoulder (9.2%) and difficulty swallowing (7.9%). Residual/recurrent malignancy or SP was confirmed in 172 (3.4%) patients at routine assessment and 51/148 (34.5%) at appointments expedited by patients.
Ilmarinen	Finland	2018	153 (366 FU visits)	FU 2014	All oropharynx, 110 (72%) p16-positive. 132 (86.3%) stage III/IV.	93 (61%) CRT, 38 (25%) surgery + RT/CRT	New symptoms that raise suspicion of cancer recurrence or patient-requested visit	4 (3%) patients developed recurrence, all of which were symptomatic. New symptom or finding reported at 26 (7.1%) appointments in 22 patients; the most common were new onset pain in pharynx or neck (10), dysphagia (3) and neck lump (3).
Lin	Taiwan	2015	136	TNO (post-treatment) 2010-2014	HNSCC plus oesophageal synchronous primaries 33.8%, hypopharynx 29.4%); remainder oropharynx, larynx and oral. 77.2% stage III/IV disease.	Majority surgery + adjuvant CRT (36.8%), primary CRT (30.9%) or surgery single modality (20.6%)	All had swallowing disorder e.g. weight loss, dysphagia, odynophagia, choking, hoarseness, prolonged tube feeding, lumping throat	45 (33.1%) patients had a recurrence/SP, all patients in cohort had swallowing disorder. No significant difference found between presence or absence of swallowing disorders and rate of local recurrence/SP. The most frequently reported swallowing disorder was dysphagia, of which 22/71 (31%) had recurrence or SP. Weight loss and odynophagia had the highest PPV (45.5% and 38.2% respectively).
Malik	India	2020	400	2018-2019	All oral cancer <2 years since treatment. 277 (69.3%) “advanced stage”, usually refers to stages III/IV.	Surgery plus 348 (87%) received adjuvant RT/CRT	Symptoms suggestive of recurrence inc. problem in speaking, new growth/ulcer in the mouth, pain in head/neck region, new neck swelling	Recurrence confirmed in 20 (5%) patients, of which the pre-clinic telephone questionnaire identified 18 (90%) however the specificity was worse than for clinical examination (75.5% versus 92.9%). Lower sensitivity for older patients, advanced disease, previous adjuvant treatment and distant recurrence.
Masroor	USA	2019	233 (3358 FU visits)	2011-2014	HPV-positive oropharyngeal SCC. 18 (7.7%) stage III disease, no stage IV.	Primary CRT (68.2%), surgery + adjuvant RT/CRT (20.6%), single modality RT or surgery	Recurrences ‘symptom-directed’ where patient symptoms prompted work-up, not pre-defined	23 recurrences, of which 11 (47.8%) - in 10 patients - were symptomatic and only 1 detected by physician examination. Remaining diagnosed on post-treatment PET-CT.
Miyamaru	Japan	2023	440	2009-2018	Oral cavity (35%), hypopharynx (29%) and larynx (28%) most common subsites. 56% stage III/IV disease.	Surgical resection +/- adjuvant RT/CRT	Patients asked about “unusual symptoms” at regular follow-up, not pre-defined	133 (30.2%) patients had 160 recurrences. 7/35 (20%) local recurrences, 17/68 (25%) regional recurrences and 5/57 (9%) of distant metastases were detected by symptom-based examination; compared with 46%, 16% and 0% with clinical examination.
Pagh	Denmark	2013	619	FU 2012	Oropharynx 199 (32%), larynx 144 (23%) and oral cavity 137 (22%) most common subsites. Stage NS.	Primary RT/CRT 419 (67.7%), surgery alone 107 (17.3%), surgery + RT 83 (13.4%)	Validated patient-reported symptom questionnaire from ESTRO, planned versus patient-requested FU	Recurrence or SP diagnosed in 29 (4.7%) patients of which 22 (75.9%) had symptoms. 19/22 (86.4%) symptomatic patients presented at routine FU. The rate of confirmed recurrence in an asymptomatic patient was 1.3%. 870 instances of HNC-related morbidity reported.
Pakkanen	Finland	2021	303	2003-2015	All T1 glottic cancer	163 (53.8%) surgery and 140 (46.2%) RT	Not pre-defined	38 (12.5%) patients had recurrence, of which 17 (44.7%) had new symptoms. The most common symptoms were hoarseness 13, pain 3 and dysphagia 3. 9 (23.7%) patients requested review due to new symptoms.
Srivastava	India	2015	86	2008-2012	Patients who still had pain at 6 weeks post-RT. 31 (36%) oropharynx, 19 (22.1%) larynx and 18 (20.9%) oral cavity most common subsites. 68 (79%) stage III/IV.	All RT/CRT	Persistent pain 6 weeks and 3 months after completion of RT	60% of patients with neuropathic-type pain at 6 weeks had a later recurrence. Among patients with persistent pain at 3m, 18 (38.3%) had a loco-regional recurrence, compared to 1 if pain was resolving at 3m.
Stimpson	Australia	2014	260 (321 FU visits)	FU 2013	NS	NS	“Presence or absence of new symptoms” not pre-defined	New symptoms reported at 59 appointments and 27 were expedited. 9 (3.5%) recurrences in total, 8 symptomatic and 1 asymptomatic. New pain was present in 3 patients (oral, neck and otalgia).
Su	USA	2018	33	2005-2016	All oropharynx HPV-positive. 10 T3/4.	NS	Not further specified	16 patients had a recurrence, 13 of whom were asymptomatic at time of recurrence detection. 12 recurrences were detected via PET-CT and none with physical examination.
Tufano-Sugarman	USA	2023	19	2011-2019	Mostly oral cavity primary (84.2%), remainder nasopharyngeal or sinus.	18 (95%) received CRT	All patients with suspected osteoradionecrosis	7 (36.8%) patients investigated for suspected ORN had cancer recurrence or persistent disease. Oral pain was the most common presenting feature of both, followed by trismus, exposed bone and mucositis/ulceration. No signs/symptoms were significantly different between ORN and recurrence. Onset of symptoms more likely to be within 6 months of treatment in recurrent/persistent SCC.
Van de Weerd	Netherlands	2024	413	2006-2012	All larynx - 264 (64%) glottic, 138 (33%) supraglottic. 132 (32%) stage III/IV.	255 (61.7%) RT/CRT, 106 (26%) surgery only, 52 (12.6%) surgery and RT/CRT	Hoarseness, change of voice, dyspnoea, dysphagia, globus sensation, pain at primary site, otalgia, bleeding or bloody sputum, neck lump	126 (30.5%) patients had recurrence, of which 98 (82%) reported symptoms at time of detection. 6 (5%) unknown symptom status. 41 (34%) were detected at an additional interval visit. Recurrence detected at an interval visit had lower 5-yr OS compared to routine (44% vs 63% p.001), however did not differ based on symptoms.
Van Nuffel	Belgium	2023	132	2005-2017	Oropharynx (70.5%), hypopharynx (29.5). 86.4% stage III/IV disease.	Primary CRT (53%), primary RT only (26.5%), single modality surgery +/- adjuvant RT/CRT	“Clinical symptoms at the time of diagnosis” of recurrence	61 (46.2%) patients had recurrence and 24 (17.8%) developed a SP tumour. 44 (72.1%) were clinically apparent or symptomatic; 11/39 (28%) patients with clinically occult recurrence had new symptoms suggestive of recurrence. Median survival in asymptomatic patients diagnosed with distant metastases was 18.5 months, compared to 4.9 months in symptomatic patients. 30% of SPs in the head and neck region were symptomatic, the same proportion as those discovered by clinical examination.
Wakasugi	Japan	2022	150	2010-2019	Oropharynx 39 (26%), hypopharynx 52 (34.7%) and oral cavity 26 (17.35) most common subsites. All stage III/IV.	Surgery plus CRT or CRT alone	“Symptoms at the time of detection of recurrence”	63 (42%) patients had recurrence and 22 (14.7%) SP tumour of which 38 (60.3%) and 8 (36.4%) were symptomatic respectively. Pain was the most common presenting symptom, reported in 20 (31.7%) cases of recurrence.
Zhang	UK	2022	1066	FU 2020	Oropharynx 426 (39.7%) and larynx 302 (28.2%) most common subsite. Includes small proportion of thyroid and skin.	NS	6m remote telephone triage and assessment of new symptoms	34 (3.2%) developed a recurrence during the study period. Locoregional recurrence found is 9.8% reporting symptoms compared to 2.3% asymptomatic. The NPV for being asymptomatic and developing recurrence was over 95% for all HNC subtypes.

FU, follow-up; NS, not stated; SCC, squamous cell carcinoma; RT, radiotherapy; CRT, chemoradiotherapy; SP, second primary; DFS, disease-free survival; TNO, trans-nasal oesophagoscopy; HPV, human papilloma virus; PCR, polymerase chain reaction; ORN, osteoradionecrosis; PPV, positive predictive value; NPV, negative predictive value; ESTRO, European Society for Therapeutic Radiology and Oncology.

### 3.3 Quality and risk of bias

The focus of the studies varied greatly, and in some cases, represent a narrow subset of the HNC patient population. For example, Pakkanen et al. only include T1 laryngeal cancer and Wakasugi et al. only include patients with locally advanced (T3/4) disease. The studies by Lin and Tufano-Sugarman, whose patients all had swallowing problems and symptoms in-keeping with osteoradionecrosis respectively, are not typical of the HNC post-treatment population. One study of patients with oropharyngeal tumours included only those who received surgical treatment, whereas the usual treatment modality for many of these patients would be primary chemoradiotherapy and therefore this is also an atypical cohort.

In most studies, the nature of patient-reported symptoms was either entirely undefined or loosely described, such as symptoms “suggestive of recurrence”. One paper used a validated questionnaire to capture patient-reported symptoms (22). We cannot be confident of the completeness of symptom data when collected retrospectively from patients’ notes although this is most pertinent for the absence of symptom data as symptoms are unlikely to have been recorded as present in error.

Seventeen studies followed patients up for an adequate length of time for recurrence to become apparent after the onset of recorded symptoms. The study by Daga et al. presented a retrospective audit of patients with HNC presenting to the hospital during a period of COVID lockdown in India for 2 months. Stimpson and colleagues invited patients attending follow-up to complete a questionnaire which included questions about the presence or absence of new symptoms. This was compared with findings at the clinic appointment, such as suspicious of recurrence. Therefore these two studies only represented a snapshot which could potentially miss recurrence associated with the reported symptoms. Since most studies were retrospective, there was not an issue with loss to follow-up or patient attrition and generally reporting bias was not a concern as all cases or consecutive cases within the period were reported.

Since most of the cohort studies were not primarily designed to evaluate symptomatic recurrence, few identified and attempted to account for associated confounding factors which could have influenced the rate of symptomatic recurrence, such as stage or treatment modality. Ten studies identified and controlled for one or more other factors which might influence the prevalence of post-treatment symptoms.

### 3.4 Results of synthesis

The diagnostic power of patient-reported symptoms to detect recurrence as reported in the 17 cohort studies are shown in **Table 2**. The median sensitivity is 47.3% [CI 44.3, 50.2], so fewer than half of patients with a recurrence will have recognisable symptoms. The reported sensitivity ranged widely from 9.1% to 100%. There was no obvious association between the predominant cancer subsites represented in the study and the sensitivity of patient-reported symptoms. Indeed, the worst reported sensitivity was in a cohort of patients all with oral cancer (13), but another study of entirely oral cancer patients by Malik et al. reported 90.0% sensitivity (19). The second worst reported sensitivity was in a study of patients with HPV-positive oropharyngeal cancer by Su et al, however the highest reported sensitivity was in the study by Ilmarinen which was also all patients with oropharyngeal cancer, of which 72% were HPV-positive. A much greater proportion of patients in the latter study were advanced stage, but again this is not a consistent pattern across the studies.

**Table 2:** Diagnostic power of patient-reported symptoms to detect recurrence.

Author (year)	N	Study design	Sensitivity (%)	Specificity (%)	PPV (%)	NPV (%)
Belcastro (2021) (12)	89	Retrospective	71.8	-	-	-
Blatt (2022) (13)	760	Retrospective	9.1	–	–	–
Brands (2022) (14)	307	Retrospective	85.2	-	-	-
Ellis (2021) (16)	5123	Retro/prospective	50.0	77.2	7.1	97.8
Ilmarinen (2019) (17)	153	Retrospective	100	87.9	18.2	100
Malik (2020) (19)	136	Prospective	90.0	75.5	16.2	99.3
Masroor (2019) (20)	233	Retrospective	47.8	-	-	-
Miyamaru (2023) (21)	440	Retrospective	18.1	–	–	–
Pagh (2013)(22)	619	Prospective	75.9	-	-	-
Pakkanen (2021) (23)	303	Retrospective	44.7	–	–	–
Srivastava (2015) (24)	86	Retrospective	94.7	56.7	38.3	97.4
Stimpson (2014) (25)	260	Prospective	88.9	83.7	13.6	99.6
Su (2018) (26)	33	Retrospective	11.5	-	-	-
Van de Weerd (2024) (28)	413	Retrospective	86.0	–	–	–
Van Nuffel (2023) (29)	132*	Retrospective	72.1	-	-	-
Wakasugi (2022) (30)	150	Retrospective	54.1	–	–	–
Zhang (2022) (31)	1066	Prospective	35.3	89.1	5.4	97.7

*Data only available for clinically occult tumours.

Sufficient data was reported in 6 cohort studies to calculate specificity, PPV and NPV of which the median is 79.3% [CI 78.3, 80.2], 9.3% [CI 7.9, 10.8] and 98.0% [CI 97.6, 98.3] respectively. This does not include case-controlled studies where either all patients were symptomatic, or all had recurrent disease. Data was able to be retrieved from studies representing all cancer subsites but only the studies by Ilmarinen and Malik included a single subsite. The specificity ranges from 56.7 - 89.1%, meaning that most patients who are asymptomatic will not have a recurrence (true negative), however symptoms are not a highly specific indicator of disease. Both PPV and NPV are determined by the prevalence of disease in the population. Five out of the six studies reported a very low recurrence rate (≤5%) and therefore NPV is expected to be high since it is inversely related to prevalence.

The most frequent patient-reported signs and symptoms varied slightly between studies but were predictable ‘red flags’ such as throat pain, hoarseness and difficulty swallowing. There is limited evidence for the relationship between the timing of patients’ symptoms and recurrence e.g. whether new onset is more pertinent. Of note, several studies report the number of patients expediting their appointments due to symptoms but there appears to be a significant cohort of patients across the studies who experienced symptoms but were seen at routine follow-up intervals.

## 4 Discussion

### 4.1 Results in context

Morbidity following treatment for HNC is common and, in the included studies, the recording of symptom rate in individuals without recurrence is poor. A European study of all cancer types (mostly breast cancer) following radiotherapy found a symptom rate of 55% and a quarter of these symptomatic patients had a recurrence (32). In comparison, the PPV of symptoms in HNC is consistently poor. This may be because the overall rate of symptoms in HNC is higher and therefore it is less discriminating for disease. A PIFU service for HNC may therefore be less efficient at detecting recurrent disease than in other cancers and have less impact in reducing follow-up demand. It also bears repeating that in PIFU programmes for other cancer types, additional testing including imaging is performed routinely (6). Since UK guidance is currently only to perform additional post-treatment imaging if clinically indicated, any such PIFU programme in HNC would need to determine on what basis imaging is requested. If it is based on symptoms then this could drive a significant increase in the demand for radiological tests.

Multiple studies found that patients with symptoms, which should have raised concern for recurrence, did not have expedited clinical review. This could be because patients did not inform their clinical team of new symptoms. If so, this is somewhat at odds with the findings from Lorenc et al. of high levels of confidence amongst HNC survivors in contacting the clinic upon identifying symptoms (33). This perhaps reflects the self-selection of patients with high levels of understanding and engagement who participated in the interview study. Patients who were interviewed also had higher rates of post-graduate education than the pool of HNC survivors they were drawn from (37.9% versus 29.5%) possibly meaning they were generally better informed. The issue of patient awareness highlights the need for patient education about red flag symptoms and self-examination. INTEGRATE audited patient education about red flag symptoms, as part of UK guidance for HNC follow-up consultations, and found documentation of verbal information-giving in 20.2% of appointments and written in 0.2% (16). This indicates significant room for improvement but could also reflect incomplete documentation.

### 4.2 Limitations of evidence

Very few studies pre-defined the symptoms which patients were asked about during follow-up. Despite this, expected symptoms of HNC recurrence such as pain and dysphagia arose repeatedly. Presumably these symptoms were elicited or volunteered by patients and recorded because they are known to be associated with HNC presentations. However, it is unclear whether this represents a complete picture of patients’ symptoms for example dry mouth, sore mouth and dental issues are very common patient-reported concerns, but these were not well represented. Most studies in this review were retrospective in nature. It is probable that there is missing data where symptoms deemed to be less important have not been recorded. This along with the lack of pre-defined symptoms means that it is not possible to draw any strong conclusions.

The patient populations in the included studies are very heterogenous; reflected in the wide range of sensitivity (9.1-100%) for patient reported symptoms. The overall prevalence of symptoms in patients after treatment for HNC is poorly described and therefore it is not possible to determine the extent to which patient-reported measures could have a meaningful role in identifying the presence of recurrence. The rate of recurrence reported in the included studies is hugely variable but particularly low for the studies which reported sufficient data to calculate PPV and NPV. Given these values are both dependent on the population prevalence, these results should be interpreted with caution.

### 4.3 Limitations of the review

Despite the terms employed in the literature search being broad, many papers were found via references and citations rather than the initial search. Regardless, the authors are confident that this approach including grey literature sources has yielded a complete picture of the available evidence on this subject. As mentioned earlier there are no valid risk-of-bias tools for observational cohort studies of this nature however we have based reporting on an existing tool and have captured the common concerns about these studies.

This review includes a heterogenous group of patients in terms of primary site, stage, treatment modality and tumour HPV-status. Tests of sensitivity and specificity are specific to the population and the population prevalence of the outcome of interest, both of which are highly variable in these studies. The tests of diagnostic power should therefore be interpreted with a significant degree of caution. This is reflected in the wide range of sensitivity and specificity values amongst the studies. This warrants further investigation with data segregated by disease subsite and with prospective collection of symptom data.

### 4.4 Implications of the results

This review suggests that symptoms in isolation are not a reliable method of detection of HNC recurrence. As patients are very unlikely to have a recurrence in the absence of symptoms, we should consider whether follow-up based primarily around recurrence detection is in patients’ best interest. Patients can have life-long morbidity after HNC diagnosis and treatment (34). For some patients, psychological morbidity including fear of cancer recurrence and body image disturbance are more pronounced than physical concerns. These aspects might be better managed in a different setting by alternative healthcare professionals e.g. Cancer Nurse Specialists (CNS) and clinical psychologists. There is a concern that PIFU models of surveillance after treatment could delay the identification of some recurrence presentations. We may question whether reliance on PIFU is justified when the outcomes of salvage treatments are generally more favourable in early stage, small volume disease.

Some studies reported that patients were experiencing symptoms at their consultation, but they were seen at a routine visit, i.e. they had not expedited their follow-up appointment. We must ensure patients are equipped with the knowledge and skills required to maximise symptom detection by educating them about red flag symptoms, empowering them to highlight to clinicians when they might be experiencing them and provide a route for urgent review.

To more accurately address the question of whether patient-reported signs and symptoms could be used to detect recurrence, patients should be asked to report all symptoms in a reliable and repeatable manner. Use of validated patient-reported outcome measures would be ideal but there should also be consensus on the measures used to compare outcomes across treatment centres. Digital tools such as smartphone applications may be useful to encourage patients to record and report signs and symptoms on a regular basis to identify symptom trends. International collaberators have already embarked on creating such as system in HNC (35). In the future, artificial intelligence tools may be trained to handle large datasets and identify common patterns which may predict recurrence however for this to be possible, accurate and more granular prospective data must be available.

## 5 Conclusion

The specificity of patient-reported symptoms is good meaning if patients do not report new or worsening symptoms, clinical teams can be reassured that the chance of recurrent disease is low. However, sensitivity is very poor in some studies therefore patient-reported signs and symptoms in isolation are not a reliable means of recurrence or SP detection. This question needs further investigation using prospective, pre-defined symptom data to build a complete picture of the prevalence of symptoms in the HNC follow-up population. Patient education, collection of data via digital symptom-tracking and the use of validated PROMs may optimise the diagnostic yield of patient-reported signs and symptoms.
