# Supplementary material for: Patient-reported symptoms in the detection of head and neck cancer recurrence: a systematic review
Source: Front Oncol. 2025 Jun 18;15:1632592. doi: 10.3389/fonc.2025.1632592 (PMC12213633; doi:10.3389/fonc.2025.1632592)
Supplement: Supplementary file 1 [file DataSheet1.docx]

**Appendix 1. Search strategy**

|  |  | **Embase 1974 to 2025 January 30**  **OvidMEDLINE 1946 to January 26 2024** |  |  |
| --- | --- | --- | --- | --- |
|  | 1 | (head and neck cancer).mp. [mp=title, book title, abstract, original title, name of substance word, subject  heading word, floating sub-heading word, keyword heading word, organism supplementary concept word,  protocol supplementary concept word, rare disease supplementary concept word, unique identifier,  synonyms, population supplementary concept word, anatomy supplementary concept word] | 116591 |  |
|  | 2 | (patient reported or patient-reported or symptom*).af. | 4220312 |  |
|  | 3 | (recurrent or recurrence or second primary).af. | 2229256 |  |
|  | 4 | 1 and 2 and 3  Embase  OvidMEDLINE | 1279  931  348 |  |

**Online repository Open Access Thesis and Dissertations (oatd.org)** searched Jan 2025 for thesis containing “head and neck cancer” (keyword) and “recurrence” in English language.

**ClinicalTrials.gov** searched Jan 2025 for “head and neck cancer” and “patient-reported symptom” for completed studies in adult patients.

**MedRxiv** searched Jan 2025 for phrase “head and neck cancer” in the 6-month period 1/1/2024-30/6/2024.

**Google search** performed Jan 2025 for “head and neck cancer recurrence patient-reported symptoms” and the first 100 hits were screened for relevance.
